# I-FABP as a Potential Marker for Intestinal Barrier Loss in Porcine Polytrauma

**DOI:** 10.3390/jcm11154599

**Published:** 2022-08-07

**Authors:** Jan Tilmann Vollrath, Felix Klingebiel, Felix Bläsius, Johannes Greven, Eftychios Bolierakis, Aleksander J. Nowak, Marija Simic, Frank Hildebrand, Ingo Marzi, Borna Relja

**Affiliations:** 1 Department of Trauma, Hand and Reconstructive Surgery, Goethe University, 60596 Frankfurt, Germany; 2 Department of Trauma, University of Zurich, Universitätsspital Zurich, 8091 Zurich, Switzerland; 3 Experimental Radiology, Department of Radiology and Nuclear Medicine, Otto von Guericke University, 39120 Magdeburg, Germany; 4 Department of Trauma and Reconstructive Surgery, RWTH Aachen University, 52074 Aachen, Germany

**Keywords:** I-FABP, biomarker, intestinal damage, hemorrhagic shock, major trauma

## Abstract

Polytrauma and concomitant hemorrhagic shock can lead to intestinal damage and subsequent multiple organ dysfunction syndrome. The intestinal fatty acid-binding protein (I-FABP) is expressed in the intestine and appears quickly in the circulation after intestinal epithelial cell damage. This porcine animal study investigates the I-FABP dynamics in plasma and urine after polytrauma. Furthermore, it evaluates to what extent I-FABP can also act as a marker of intestinal damage in a porcine polytrauma model. Eight pigs (Sus scrofa) were subjected to polytrauma which consisted of lung contusion, tibial fracture, liver laceration, and hemorrhagic shock followed by blood and fluid resuscitation and fracture fixation with an external fixator. Eight sham animals were identically instrumented but not injured. Afterwards, intensive care treatment including mechanical ventilation for 72 h followed. I-FABP levels in blood and urine were determined by ELISA. In addition, immunohistological staining for I-FABP, active caspase-3 and myeloperoxidase were performed after 72 h. Plasma and urine I-FABP levels were significantly increased shortly after trauma. I-FABP expression in intestinal tissue showed significantly lower expression in polytraumatized animals vs. sham. Caspase-3 and myeloperoxidase expression in the immunohistological examination were significantly higher in the jejunum and ileum of polytraumatized animals compared to sham animals. This study confirms a loss of intestinal barrier after polytrauma which is indicated by increased I-FABP levels in plasma and urine as well as decreased I-FABP levels in immunohistological staining of the intestine.

## 1. Introduction

Despite significant achievements that reduced injury-related morbidity and mortality in recent decades, the number of polytraumatized patients admitted to hospitals still remains high and worldwide approximately 4.8 million human deaths per year are caused by traumatic injuries [1]. After traumatized patients survive the first phase after injury, they are at high mortality risk from late-occurring complications such as multiple organ failure (MOF) and/or sepsis [2,3]. For several decades, the gut has been considered to play an important role in the development of systemic inflammation, sepsis and multiple organ dysfunction syndrome (MODS) after trauma, hemorrhagic shock and in critically ill patients in general [4,5]. The predominant theory in the 1980s associating the gut with MODS underlines the key role of bacterial translocation caused by an increased intestinal hyperpermeability allowing bacteria to enter the portal blood and cause downstream organ dysfunction [5,6]. In contrast, more recent studies emphasize the role of the mesenteric lymph as a carrier of gut-derived danger-associated molecular patterns (DAMPs) to the lung and the systemic circulation, leading to the “gut-lymph hypothesis” [6,7]. Furthermore, recent data suggest that gut luminal contents, including the mucus gel layer, pancreatic proteases and gut flora, as well as the luminal response to splanchnic ischemia, play an important role in modulating gut injury [4]. Regardless of the underlying mechanisms, early recognition of patients with intestinal injury and at risk of developing MODS or MOF is of enormous clinical relevance. D-lactate, glutathione S-transferase (GST) and intestinal fatty acid binding protein (I-FABP) have been proposed as novel biomarkers of intestinal ischemia, and plasma citrulline levels have been proposed as a novel quantitative biomarker of significantly reduced enterocyte mass and function [4,8]. FABPs are small proteins localized intracellularly or within the plasma membrane and are released in their soluble form into the extracellular space early after cell or tissue damage [9,10]. Therefore, FABPs can be used as urine and plasma markers for tissue-specific injuries [9,11]. I-FABP is present in enterocytes of the small intestine as well as partly in the colon and appears quickly in the circulation after intestinal epithelial cell damage [9,12]. Voth et al. investigated I-FABP levels in polytraumatized patients and confirmed I-FABP as a useful and promising early marker for the detection of abdominal injury [9,13]. Furthermore, even in the absence of an abdominal injury, I-FABP indicated intestinal damage by hemorrhagic shock [9]. In this study, we investigated I-FABP plasma and urine levels in a standardized porcine trauma model which consisted of lung contusion, tibial fracture, liver laceration, and hemorrhagic shock followed by fluid resuscitation and fracture fixation with an external fixator. Furthermore, this study provides insights into changes in I-FABP-, caspase-3- and myeloperoxidase levels in intestinal tissue after polytrauma which are not possible to be investigated in human clinical studies.

## 2. Materials and Methods

### 2.1. Ethics

All experiments were conducted in compliance with federal German law. Especially with regard to the protection of animals, institutional guidelines and the criteria in “Guide for the Care and Use of Laboratory Animals” (Eighth Edition The National Academies Press, 2011) [14]. All animals were continuously treated in consensus with the ARRIVE guidelines [15]. Experiments were authorized by the responsible government authority (“Landesamt für Natur-, Umwelt- und Verbraucherschutz”: LANUV-NRW, Germany, AZ: 81.02.04.2018.A113). The in vivo experiments were performed at the Institute of Laboratory Animal Science & Experimental Surgery, RWTH Aachen University, Germany.

### 2.2. Animals

16 German landrace pigs (*Sus scrofa*, 30 ± 5 kg, male, three months old) from a disease-free barrier breeding facility were used for the experiment. Before the beginning of the experiment, the animals were fasted overnight but had free access to water ad libidum. The animals were held in rooms with air-conditioning and had the opportunity to acclimatize to the new environment for a minimum of seven days before the experiment started. Furthermore, animals were examined by a veterinarian ahead of the beginning of the experiment. The porcine polytrauma model is well established in our study group and has been reported in detail by Horst et al. [16]. The underlying study was part of a larger animal study containing polytraumatized animals and sham animals that were not injured.

### 2.3. Porcine Polytrauma Model

**Figure 1:**
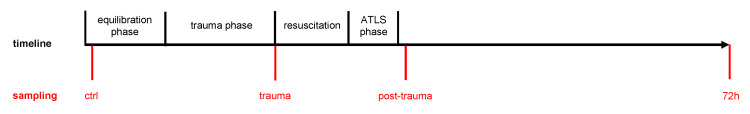
The experimental design is shown. Animals were subjected to polytrauma which consisted of lung contusion, tibial fracture, liver laceration and hemorrhagic shock. Polytrauma was followed by blood and fluid resuscitation as well as fracture fixation with an external fixator. Blood sampling was performed at the beginning shortly after implementation of the central venous catheter (ctrl), shortly after trauma (trauma) and in the further course after the Advanced Trauma Life Support (ATLS) phase (post-trauma).

A total of eight pigs were attributed to a standardized polytrauma which consisted of a tibia fracture, a liver laceration, a unilateral blunt chest trauma and hemorrhagic shock (40 ± 5 mm Hg for 90 min). During the following, ATLS-phase animals were resuscitated and the fracture was fixated with the help of an external fixator. Eight sham animals underwent identical instrumentation but were not traumatized. In advance of the beginning of the experiment, all animals received an intramuscular application of 4 mg/kg azaperone (Stressni™, Janssen, Neuss, Germany). Before surgery and every 24 h during the further experiment a prophylactic antibiotic treatment was administered (1.5 g cefuroxime i.v., Fresenius Kabi, Bad Homburg, Germany). In order to induce anesthesia, propofol was injected into the vein at a dosage of 3 mg/kg (Propofol Claris 2% MCT, Pharmore GmbH, Ibbenbüren, Germany). After that, pigs were intubated orotracheally with a 7.5 ch tube (Hi-Lo Lanz™, Medtronic, Meerbusch, Germany). In order to prevent awareness and pain during the further course of the experiment, general anesthesia was sustained with intravenous application of propofol, fentanyl (Rotexmedica, Trittau, Germany) and midazolam (Rotexmedica, Trittau, Germany). During the whole experiment, pigs were ventilated with lung protective ventilation (Draeger, Evita 4, Lübeck, Germany). The tidal volume was set to 6–8 mL/kg. Furthermore, positive end-expiratory pressure (PEEP) was set to 8 mm Hg (plateau pressure < 28 mm Hg) and arterial pCO_2_ was aimed to be between 35 and 45 mm Hg which was regularly controlled with the help of blood gas analysis. To maintain anesthesia, and monitor central venous pressure and provide fluids, a sterile central venous catheter (4-Lumen Catheter, 8,5 Fr, Arrow Catheter, Teleflex Medical, Fellbach, Germany) was inserted into the right external jugular vein. Furthermore, a sterile two-lumen hemodialysis catheter (Arrow International, Teleflex Medical, Fellbach, Germany) was implemented into the left femoral vein to generate hemorrhagic shock adequately in the further experimental course. To monitor blood pressure and to generate arterial blood gas analysis an arterial catheter (PiCCO, Pulsion Medical Systems, Feldkirchen, Germany) was inserted into the right femoral artery. Moreover, a urinary catheter was inserted into the bladder (12.0 Fr, Cystofix, Braun, Melsungen, Germany). The first blood samples were taken shortly after the implementation of the central venous catheter. Before subjecting the pigs to polytrauma, an equilibration period of one hour was awaited. The porcine polytrauma was induced as previously described by Horst et al. but with slight modifications [16]. To represent the situation and condition during trauma more closely the fraction of inspired oxygen (FiO_2_) was set to 21% before the initiation of trauma. Furthermore, fluid administration was restricted to a minimum of 10 mL/h. The endeavors to maintain normothermia with the help of forced-air warming systems were stopped and a drop in body temperature into hypothermia was not prevented anymore to imitate the preclinical situation. Animals were placed on the left side and the tibia was fractured with the help of a bolt gun (Blitz-Kerner, turbocut JOBB GmbH, Bad Neustadt an der Saale, Germany; Ammunition: 9 × 17 mm^2^, RUAG Ammotec GmbH, Fürth, Germany). Then pigs were placed on the right side followed by induction of blunt thoracic trauma with the help of a bolt shot to the left dorsal lower thorax. After that, a midline-laparotomy was performed and the caudal lobe of the liver was incised (4.5 × 4.5 cm^2^) leading to uncontrolled bleeding. Bleeding was stopped after 30 s by packing with five sterile gauze compresses (10 × 10 cm^2^). To induce hemorrhagic shock, blood was withdrawn from the femoral venous catheter until a mean arterial blood pressure (MAP) of 40 ± 5 mm Hg. This state of hemorrhagic shock was maintained for 90 min. Resuscitation was performed by returning the withdrawn blood, adjusting the FiO_2_ to baseline and infusion of crystalloid fluids (4 mL/kg body weight/h). To reach normothermia (38.7–39.8 °C) again, warm air was applied with the help of a forced-air warming system. The tibial fracture was fixated with the help of an external fixator. Further intensive care treatment, as well as the management of complications (e.g., pneumothorax), were performed according to the latest recommendations of the European Resuscitation Council and the Advanced Trauma Life Support (ATLS^®^) [17,18]. After 72 h pigs were euthanized with the help of potassium chloride. Figure 1 shows an overview of the experimental design.

### 2.4. Blood and Urine Sampling and Processing

Blood samples were obtained shortly after the implementation of the central venous catheter (ctrl), shortly after trauma (trauma) and in the further course after the ATLS phase (post-trauma) in prechilled ethylendiaminetetraacetic acid tubes (EDTA, S-Monovette^®^, Sarstedt, Nümbrecht, Germany) and kept on ice. Then, blood samples were centrifuged at 2000× *g* for 15 min at 4 °C and the supernatant was stored at −80 °C until further analysis. The same protocol was applied for urine samples.

### 2.5. ELISA Measurements

I-FABP ELISA measurements were performed with plasma and urine samples in accordance with the manufacturer’s manuals (Porcine IFABP/FABP2 (Intestinal Fatty Acid Binding Protein) ELISA Kit, #MBS2501296, MyBioSource, Inc., San Diego, CA, USA). According to the manufacturer the used I-FABP ELISA Kit has a sensitivity is 46.88 pg/mL. Before ELISA blood and urine were centrifuged at 2000× *g* for 15 min at 4 °C and supernatant was stored at −80 °C until further analysis.

### 2.6. Immunohistochemical Staining of Intestine Tissue

Parts of jejunum and ileum (1 × 1 cm^2^) were stored in 4% formalin for overnight fixation and kept in 70% ethanol until paraffin embedding and sectioning. Paraffin-embedded intestine samples were sectioned (3 µm), deparaffinized, rehydrated and stained with anti-FABP2 antibody (Cloud-Clone Corp., Houston, TX, USA), anti-Myeloperoxidase antibody (Abcam, Cambridge, UK) or anti-Caspase-3 antibody (Abcam, Cambridge, UK). Following deparaffinization, antigen retrieval was conducted under a steam atmosphere using an R-Universal epitope recovery buffer (Aptum, Kassel, Germany) for one hour (Retriever 2010, Prestige Medical). The endogenous tissue peroxidase activity was blocked with hydrogen peroxide according to the manufacturer’s instructions (Peroxidase UltraVision Block, Dako, Hamburg, Germany). After washing with water and PBS, anti-FABP2 antibody (1:100, PAA559Po01), anti-Myeloperoxidase antibody (1:100, ab9535) or anti-Caspase-3 antibody (1:100, ab4051) was applied as a primary antibody. After the incubation at room temperature for one hour, and a subsequent washing procedure, a secondary anti-rabbit horseradish peroxidase-linked antibody (Nichirei Biosciences Inc., Tokyo, Japan) was applied to detect specific binding. As the substrate, 3-amino-9-ethylcarbazole (AEC, DCS Innovative Diagnostik-Systeme, Hamburg, Germany) was applied. Then, the sections were counterstained with hematoxylin. The relative staining intensity of the AEC substrate per slide was evaluated using the ImageJ software in a blinded manner by an independent examiner for anti-FABP2 staining. For MPO and caspase-3 15–20 high power fields per slide were quantified by counting positively stained cells.

### 2.7. Statistical Analyses

Statistical analyses were performed by using GraphPad Prism 6 (Graphpad Software Inc., San Diego, CA, USA). Data are presented as mean ± standard error of the mean. Based on the D’Agostino–Pearson normality test differences between the groups were determined by the non-parametric Kruskal–Wallis test followed by Dunn’s post hoc test for the correction of multiple comparisons. The comparison between polytrauma and sham groups was performed using the unpaired Mann–Whitney test. A *p*-value less than 0.05 was considered to be statistically significant.

## 3. Results

### 3.1. Data Decription

#### 3.1.1. Plasma and Urine I-FABP Concentrations after Trauma

**Figure 2:**
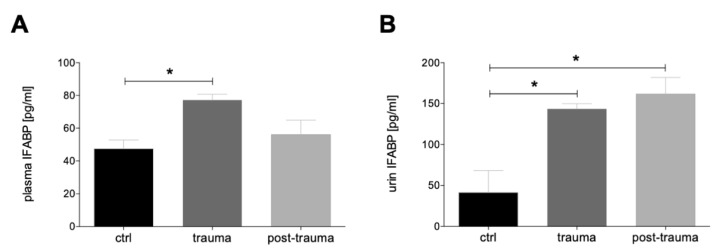
Intestinal fatty acid binding protein (I-FABP) levels in plasma and urine. Plasma (**A**) and urine (**B**) I-FABP levels shortly after implementation of the central venous catheter (ctrl), shortly after trauma (trauma, within 90 min during trauma and hemorrhagic shock) and in the further course after the Advanced Trauma Life Support (ATLS) phase (post-trauma, within 6 h after reperfusion and surgery) are shown. *: *p* < 0.05.

The time course of I-FABP concentration in the blood shows a significant increase shortly after trauma compared to the control before trauma (*p* < 0.05). In the further posttraumatic course, I-FABP concentration in the blood decreases again reaching the pre-trauma ctrl levels (Figure 2A). The time course of urine I-FABP-concentration shows a distinct and statistically significant increase shortly after trauma and in the further posttraumatic course compared with the ctrl (*p* < 0.05, Figure 2B).

#### 3.1.2. I-FABP Expression in Intestinal Tissue

**Figure 3:**
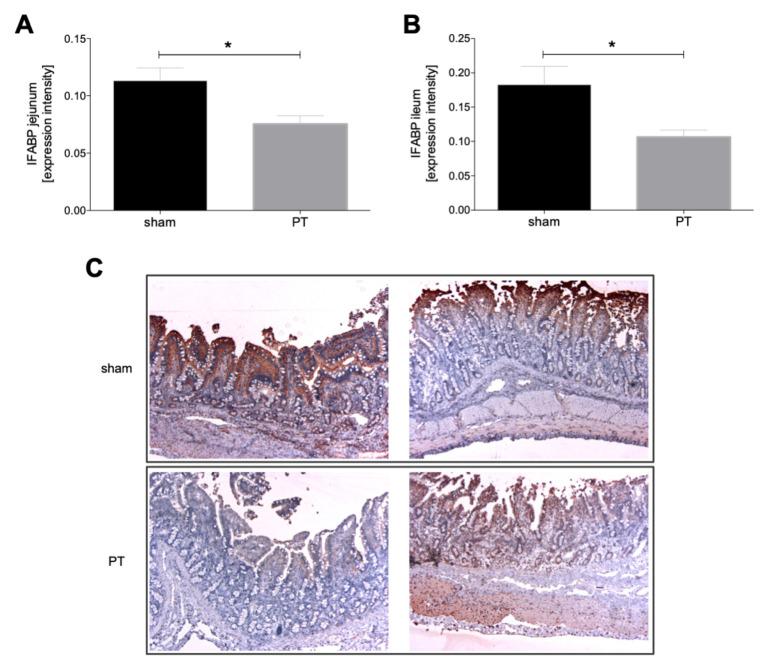
Expression intensity of intestinal fatty acid binding protein (I-FABP) in jejunum (**A**) and ileum (**B**) in polytraumatized (PT) and sham animals after 72 h is shown. (**C**) Exemplary images of immunohistological staining for I-FABP of ileum and jejunum from polytraumatized and sham animals. *: *p* < 0.05.

A comparison of the I-FABP expression in the intestinal tissue of sham and polytraumatized animals shows significantly lower I-FABP expression in the jejunum of polytraumatized animals compared to sham animals (*p* < 0.05, Figure 3A). The same significantly decreased expression after polytrauma vs. sham can be observed in the ileum (*p* < 0.05, Figure 3B). Figure 3C shows exemplary histological I-FABP stainings from the jejunum and ileum in sham and polytraumatized animals.

#### 3.1.3. Caspase-3 Expression in Intestinal Tissue

**Figure 4:**
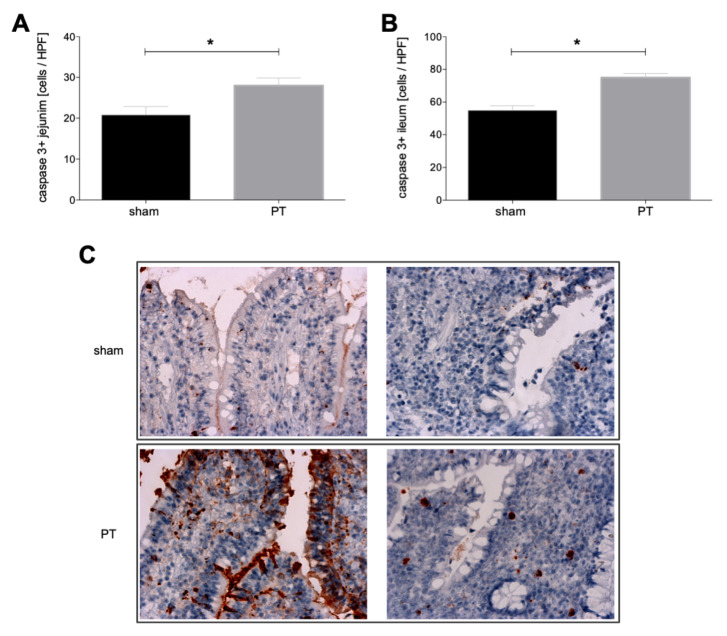
Number of caspase-3 positive cells per high power field (HPF) in jejunum (**A**) and ileum (**B**) of polytraumatized (PT) and sham animals after 72 h is shown. (**C**) Exemplary images of immunohistological staining for caspase-3 of ileum and jejunum from polytraumatized and sham animals. *: *p* < 0.05.

A comparison of the active caspase-3 expression in intestinal tissue from sham and polytraumatized animals shows significantly higher expression in the jejunum of polytraumatized animals compared to sham animals (*p* < 0.05, Figure 4A). Significantly increased expression after polytrauma vs. sham can be observed in the ileum (*p* < 0.05, Figure 4B). Figure 4C shows exemplary histological caspase-3 stainings from the jejunum and ileum in sham and polytraumatized animals.

#### 3.1.4. Myeloperoxidase Expression in Intestinal Tissue

**Figure 5:**
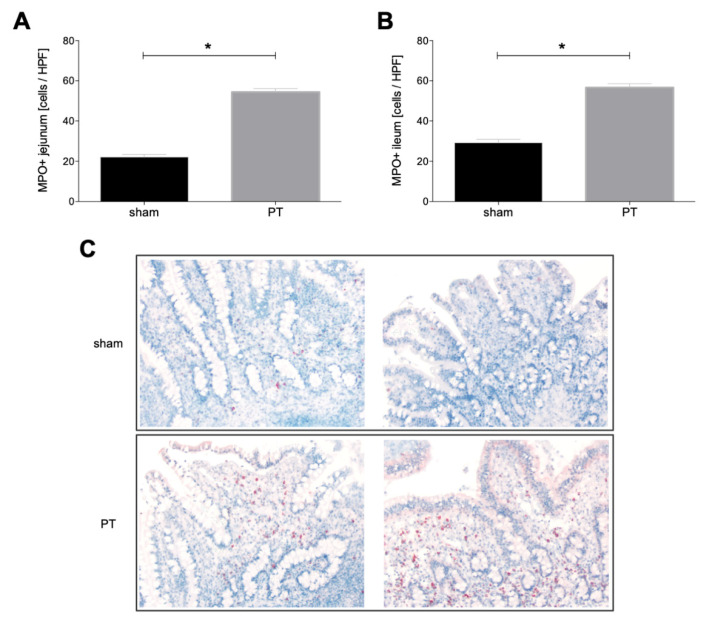
Number of myeloperoxidase positive cells (MPO^+^) per high power field (HPF) in jejunum (**A**) and ileum (**B**) of polytraumatized (PT) and sham animals after 72 h is shown. (**C**) Exemplary images of immuno-histological staining for MPO of ileum and jejunum from polytraumatized and sham animals. *: *p* < 0.05.

A comparison of the myeloperoxidase expression in intestinal tissues from sham and polytraumatized animals shows significantly higher expression in both jejunum and ileum of polytraumatized animals compared to sham animals (*p* < 0.05, Figure 5A,B).

## 4. Discussion

In the recent study, we investigated plasma and urine I-FAPB levels in a standardized porcine polytrauma model and observed significantly increased plasma I-FABP levels immediately after trauma while plasma I-FABP decreased in the further course. This is in line with studies by Voth et al. showing increased I-FABP levels already in the emergency room in polytraumatized patients with concomitant abdominal injury, as well as in polytraumatized patients with hemorrhagic shock [9,13]. Voth et al. observed I-FABP levels decreasing to control levels already on day one after trauma [9,13]. Khadaroo et al. investigated I-FABP as a biomarker for the early diagnosis of acute mesenteric ischemia in mice and observed significantly increased plasma I-FABP levels already after ischemia of 30 min resulting in a further increase with values continuing to rise clearly with increasing ischemia time [19]. Furthermore, a significant rise in plasma I-FABP concentrations could already be demonstrated 15–30 min after clamping of the mesenteric artery in a porcine ischemia model [20]. Several other studies report elevated serum I-FABP levels in patients with mesenteric ischemia [21,22,23]. In line with these studies which observed increased levels of I-FABP in the blood in the context of an undersupply of the intestine, we assume that in our polytrauma model an undersupply of the intestine with blood probably occurs in the context of the hemorrhagic shock phase. This theory is supported by the underlying data showing significantly more caspase-3 positive cells in the intestine of polytraumatized animals, which corresponds to an increased apoptosis rate. However, since our study does not have a separate group with hemorrhagic shock without further injuries, this can only be speculated. In line with our results, Hotchkiss et al. reported rapidly occurring apoptosis in lymphocyte and intestinal epithelial cells in patients with trauma and ischemia/reperfusion injury and concluded that their results support the theory that gut mucosal apoptosis may compromise bowel integrity and lead to translocation of bacteria and endotoxin into the systemic circulation [24].

In line with this, we observed decreased I-FABP expression in the small intestine and increased I-FABP plasma levels in animals that were exposed to multiple traumas, which could be an indication of possible damage to the intestinal barrier. Sturm et al. also observed a significant increase in I-FABP in serum following acute alcohol consumption and concluded that this might suggest possible damage to the intestinal barrier [25]. In line with the study by Khadaroo et al. showing increased serum I-FABP levels as well as increased MPO activity in the intestine after mesenteric ischemia [19], we also observed increased MPO activity in the intestine after polytrauma. Furthermore, Khadaroo et al. observed that intestinal ischemia resulted in lung injury in a time-dependent manner and that I-FABP also directly correlated with resultant lung injury [19]. The potential link between the gut and the lung might be the mesenteric lymph as shown by Lu et al. [26]. In an animal model the authors could demonstrate that trauma-hemorrhagic shock induces pulmonary endothelial cell apoptosis and that pulmonary endothelial and nonendothelial cell apoptosis occurs largely due to gut-derived factors carried in the mesenteric lymph [26]. Nowadays it is undisputed that intestinal barrier dysfunction and increased gut permeability are associated with the development of MODS [7]. While clinical data did not confirm the initial theory of bacterial translocation, the current pathogenetic aspects support the gut-lymph axis which is reviewed in detail elsewhere [7]. Anyway, the gut seems to be a pivotal proinflammatory organ promoting deleterious effects in even remote organs, through the release of DAMPs, without the need for systemic bacterial translocation [7]. However, what both theories have in common is the loss of the intestinal barrier, which was also shown in this study by lower I-FABP expressions in the immunohistological staining of the intestine as well as increased blood and urine I-FABP levels after trauma.

It must be mentioned and considered that this animal study has several limitations. First of all, and of utmost importance, is the limited sample size which is due to animals’ welfare and the very high cost of large animal models. Moreover, due to the limited duration of the experiment, we cannot make a well-founded statement about whether the observed changes in the intestinal barrier will influence the outcome beyond the observational period of 72 h. Furthermore, we must be aware of the immunological variability among different species, when we transfer findings from animal experiments to humans [27]. Another limitation of the study is that it only indicates a loss of the intestinal barrier after polytrauma. It is purely observational, without investigating the pathogenesis and does not answer the question of which aspect of the traumatic injury (hemorrhagic shock, fracture, lung injury, etc.) leads to the disruption of the intestinal barrier. Furthermore, additional biochemical or observational markers of severity have not been associated with I-FABP levels. However, in this regard, we need to consider the original characterization study of the underlying model, where a descriptive assessment of inflammatory changes has been performed. Horst et al. have described an early systemic increase in inflammatory parameters with a decline during the time course after polytrauma [16]. In another study in polytraumatized patients, we have shown that I-FABP correlated with IL-6 und PCT levels. That study revealed the early presence of intestinal epithelial cell damage in trauma patients. The extent of intestinal damage was associated with the presence of shock and injury severity. Early intestinal damage preceded and was related to the subsequent developing inflammatory response [28]. In the underlying study, we measured IL-6 at 72 h after polytrauma in the samples that still were available. We found very low but still detectable levels of IL-6 by using a high-sensitivity ELISA kit. The levels of IL-6 were statistically significantly different between sham and polytrauma (1.685 ± 0.389 vs. 3.387 ± 0.532 pg/mL, *p* = 0.030) after 72 h. Taken together, according to that and the studies reported above, I-FABP increase appears associated with an increase in inflammatory parameters in the underlying model. Another limitation of the study is the administration of medications such as antibiotics, opiates, or the lack of enteral nutrition during the experiment which can lead to a distortion of the results. Furthermore, the quantification of I-FABP expression is a bit arbitrary, and in future studies, western blot analyses, as well as additional markers of enterocyte damage, should be implemented. Moreover, since we did not do other tissue-specific analyses of FABP, we cannot exclude that this array cross-reacts with FABP from origins other than the intestine. Another limitation is certainly I-FABP itself which is still arguably a biomarker without clinical applicability. Timmermans et al. investigated intestinal damage using I-FABP in trauma patients during the first days of their hospital admission and observed substantial differences in plasma I-FABP levels between patients with abdominal trauma and low Hb/MAP and patients with other trauma types and normal/high Hb/MAP [29]. Therefore, the authors concluded that targeted interventions, such as (more aggressive or more goal-directed) fluid resuscitation and/or hemodynamic support, may represent a viable treatment option in these subgroups of patients. Because the intestinal injury is suggested to be related to late complications, such as MODS or sepsis in trauma patients, strategies to prevent intestinal damage consequences or the persisting damage itself after trauma could be of benefit to these patients. Yet, clinicians can also make decisions on the amount of volume resuscitation or hemodynamic support based on other clinical or technical parameters as well. Measurement of I-FABP can only provide an additional read-out parameter. Voth et al. showed that I-FABP levels are significantly increased in patients with an intestinal injury compared to patients with abdominal injury but without intestinal damage and concluded that I-FABP might be a useful and promising early marker for the detection of an injury to the small or large intestine [13]. Thus, a threshold of the initial I-FABP level indicating the need for exploratory surgery would be of significant clinical importance. Thus, it is of utmost clinical importance to elaborate on whether I-FABP allows early diagnosis of specific intestinal injuries that cannot be seen on CT scan and therefore may be overlooked.

## 5. Conclusions

In conclusion, our study indicates a loss of intestinal barrier after polytrauma which is demonstrated by increased I-FABP levels in plasma and urine, as well as decreased I-FABP levels in immunohistological staining of the intestine. Future studies in larger cohorts need to investigate the pathogenesis, the underlying molecular pathways and possible treatment options.

## Data Availability

The data are available upon a reasonable request from the corresponding author.
